# Annually resolved North Atlantic marine climate over the last millennium

**DOI:** 10.1038/ncomms13502

**Published:** 2016-12-06

**Authors:** D. J. Reynolds, J. D. Scourse, P. R. Halloran, A. J. Nederbragt, A. D. Wanamaker, P. G. Butler, C. A. Richardson, J. Heinemeier, J. Eiríksson, K. L. Knudsen, I. R. Hall

**Affiliations:** 1 School of Earth and Ocean Sciences, Cardiff University, Main Building, Park Place, Cardiff CF10 3AT, UK; 2 School of Ocean Sciences, College of Natural Science, Bangor University, Menai Bridge, Anglesey LL59 5AB, UK; 3 Department of Geography, College of Life and Environmental Sciences, University of Exeter, Exeter EX4 4RJ, UK; 4 Department of Geological and Atmospheric Sciences, Iowa State University, Ames, Iowa 50011-3212, USA; 5 Aarhus AMS Centre, Department of Physics and Astronomy, Aarhus University, Ny Munkegade 120, DK-8000 Aarhus C, Denmark; 6 Institute of Earth Sciences, University of Iceland, Askja, Sturlugata 7, IS-101 Reykjavík, Iceland; 7 Department of Geoscience, Aarhus University, Høegh-Guldbergs Gade 2, DK-8000 Aarhus C, Denmark

## Abstract

Owing to the lack of absolutely dated oceanographic information before the modern instrumental period, there is currently significant debate as to the role played by North Atlantic Ocean dynamics in previous climate transitions (for example, Medieval Climate Anomaly-Little Ice Age, MCA-LIA). Here we present analyses of a millennial-length, annually resolved and absolutely dated marine δ^18^O archive. We interpret our record of oxygen isotope ratios from the shells of the long-lived marine bivalve *Arctica islandica* (δ^18^O-shell), from the North Icelandic shelf, in relation to seawater density variability and demonstrate that solar and volcanic forcing coupled with ocean circulation dynamics are key drivers of climate variability over the last millennium. During the pre-industrial period (AD 1000–1800) variability in the sub-polar North Atlantic leads changes in Northern Hemisphere surface air temperatures at multi-decadal timescales, indicating that North Atlantic Ocean dynamics played an active role in modulating the response of the atmosphere to solar and volcanic forcing.

## Abstract (web summary)

A lack of annually resolved climate records from the marine archive limits our understanding of oceanic processes. Here, the authors present a millennial-length, annually-resolved and absolutely-dated marine δ^18^O record from the shells of marine bivalves and offer insight into North Atlantic climate dynamics.

The climate of the last 1,000 years is characterized by the gradual pre-industrial cooling of the Northern Hemisphere associated with the Medieval Climate Anomaly-Little Ice Age (MCA-LIA) transition^1^ and the onset of modern warming^2^. Over these timescales, natural temperature variability has been linked to interactions between fluctuations in total solar irradiance (TSI)^2^,^3^, volcanic aerosols^2^,^4^ and internal climate system mechanisms (for example, ocean heat storage and transport^5^). With climate model simulations suggesting a significant slowdown in the Atlantic Meridional Overturning Circulation, in response to anthropogenic forcing over the twenty-first century^6^, there is a pressing need to provide a more robust quantitative assessment of the role changes in the North Atlantic Ocean dynamics have played in the evolution of the climate system during the recent past.

Our current understanding of past climate fluctuations is largely derived from instrumental time series^7^,^8^, numerical climate models (for example, see ref. 9) and robustly dated well-calibrated proxy archives (for example, dendrochronologies^10^,^11^, ice cores^12^,^13^, corals^14^ and speleothems^15^) at high frequencies (inter-annual) and ocean sediment archives at lower resolutions (multi-decadal to centennial)^16^,^17^. These records have highlighted the inherent complexities in the ocean–atmosphere system with TSI^3^ and volcanic aerosols^2^,^4^,^18^ acting as top–down forcings driving ocean and atmosphere variability, coupled with internal variability and feedback mechanisms within the ocean acting as bottom-up drivers modulating the atmospheric climate response^19^. However, the interaction between ocean dynamics (such as sea surface temperature (SST), seawater density, surface circulation and overturning circulation strength) and atmospheric variability remain poorly characterized. The difficulty in constraining the role of coupled ocean–atmosphere mechanisms in climate variability to a large extent results from the limited length of oceanographic instrumental time series^7^,^8^ and the low temporal resolution and dating uncertainties associated with marine sediment archives. These issues hinder the identification of causal relationships through for example lead–lag analysis. Despite these challenges, evidence derived from marine proxy records has indicated that there were broad scale changes in the North Atlantic dynamics over the last millennium. For example, during the MCA-LIA transition, analyses of foraminifera from the Straits of Florida indicate the strength of the Gulf Stream and associated heat transport reduced by *ca.* 10% (ref. 20). Similarly, regional marine radiocarbon reservoir (Δ*R*) determinations from long-lived bivalves suggest an increase in the proportion of Arctic water entrained onto the North Icelandic shelf^21^ at this time and reconstructions derived from coralline algae^22^ and sedimentary biomarker records^23^ suggest a synchronous increase in Arctic and Icelandic sea-ice extent. Finally, radiocarbon determinations from deep sea benthic foraminifera also indicate changes in the deep-water composition of the Atlantic^24^, coincident with these upper ocean shifts. These studies demonstrate that variability across the North Atlantic marine environment was synchronous, within the temporal uncertainties, with the gradual reduction in Northern Hemisphere surface air temperature (NHSAT) over the MCA-LIA. However, the resolution and lack of absolute dating of all of these proxy records preclude the assessment of whether variability in the marine environment was playing an active role in driving the changes in NHSAT over the MCA-LIA transition, because they are not suitable for detailed lead–lag analysis. These constraints present a clear challenge to understanding the role ocean dynamics play in the wider climate system.

Here we report a new 1,048-year precisely dated, annually resolved marine oxygen isotope record (δ^18^O-shell) that spans the entirety of the last 1,000 years (AD 953–2000). This high-resolution record facilitates lead–lag analysis of high-frequency climate variability occurring across the ocean and atmosphere beyond the observational era. The δ^18^O-shell record is derived from the δ^18^O composition of aragonite sampled from the annually formed growth increments in the shells of the long-lived marine bivalve mollusc *Arctica islandica* collected from the North Icelandic shelf (66° 31.59′ N, 18° 11.74′ W; shells collected from 80 m water depth^25^; Fig. 1). This location and water depth situates the shells within the inner North Icelandic Irminger Current (NIIC; Fig. 1). This oceanographic setting is ideally situated for the examination of the role that ocean dynamics play in driving wider climate variability, reflecting the interplay between two distinct water masses: the relatively warm and saline Subpolar Mode Water (SPMW) and the cool and fresh Arctic Intermediate Water (AIW). Approximately 5–10% of the ∼10–19 Sv (1 Sv=10^6^ m^3^ s^−1^) of SPMW water and associated heat flux, transported north in the Irminger Current, flowing as part of the sub-polar gyre, separates in the southern Denmark Strait and forms the clockwise flowing NIIC (Fig. 1 and refs 26, 27). As the NIIC flows along the North Icelandic shelf large amounts of polar AIW waters are entrained, increasing the net volume transport of the outermost branch of the NIIC from 1.1 to 2 Sv (55% SPMW/45% AIW)^27^. However, the somewhat weaker (∼0.3 Sv) inner NIIC retains its SPMW characteristics as it follows the inner North Icelandic shelf eastward at water depths of up to ∼100 m (Fig. 1c)^27^.

## Results

### Oxygen isotope time series

**Figure 2:**
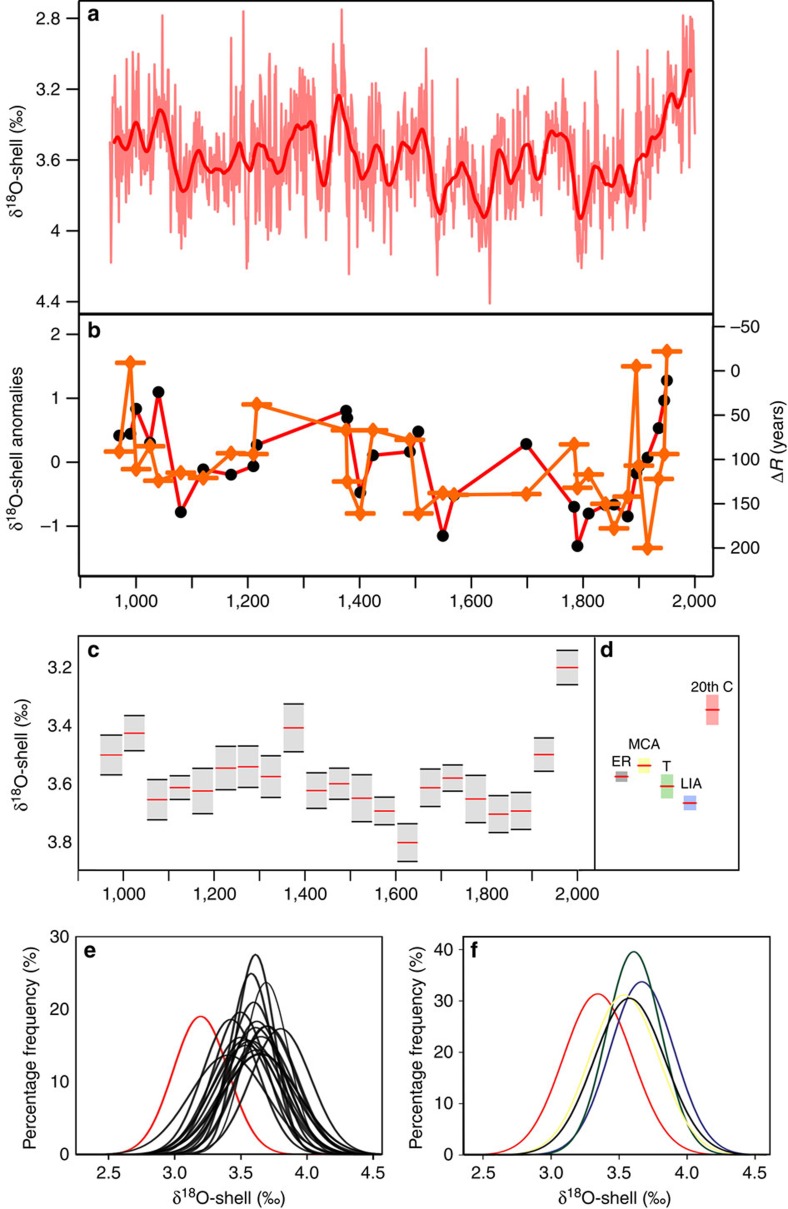
The δ^18^O-shell series and ΔR record. (**a**) The annually resolved δ^18^O-shell series (pink line) plotted with a 30-year loess first-order low-pass filter (thick red line). (**b**) The North Icelandic marine reservoir age series^21^ (orange line with diamonds and bar) and the δ^18^O-shell values for the corresponding years (red line with black dots). The δ^18^O-shell series was low-pass filtered to match the temporal resolution of the Δ*R* record. (**c**) Mean δ^18^O-shell values plotted with associated 95% confidence intervals calculated over 50-year bins (with zero years overlap) and (**d**) mean δ^18^O-shell values calculated over the twentieth century (20th C; red bar), the Little Ice Age (LIA; blue bar), the MCA-LIA transition (T; green Bar), the Medieval Climate Anomaly (MCA; yellow bar) and the entire isotope record (ER; grey bar). (**e**,**f**) Percentage frequency distributions of the δ^18^O-shell record calculated over (**e**) 50-year bins (red line corresponds to 1951–2000, whereas the black lines correspond the previous 50-year bins) and (**f**) the twentieth century (red line), the Little Ice Age (blue line), the MCA-LIA transition (green line), the Medieval Climate Anomaly (yellow line) and the entire isotope record (black line).

The age of each δ^18^O sample comprising the 1,048-year δ^18^O-shell record is derived from an absolutely dated growth increment width master chronology constructed using cross-dating methods derived from dendrochronology^21^,^28^. In total, the δ^18^O-shell series is constructed from the analysis of 1,492 samples reflecting either single δ^18^O measurements from individual years within the growth increment width master chronology or the arithmetic mean in years represented by replicate sample analyses (Supplementary Fig. 2). The growing season of the *A. islandica* shells is defined using sub-annually (sub-incremental) resolved δ^18^O analyses over the period 1960–1976 (Supplementary Fig. 3). Examination of the sub-annually resolved δ^18^O analyses relative to local instrumental seawater temperature (SWT) observations and the relative sampling position within each increment indicates that 60–80% of the shell growth takes place between June and late September each year, with slower rates of growth occurring during spring (April to June) and autumn (September to October). The annually resolved δ^18^O-shell series (Fig. 2) contains a gradual increase in δ^18^O ratios over the period AD 953–1891 (±18 years)^29^ after which there is a rapid transition to lower values. Examination of the mean and percentage frequency distribution of these data over the key periods of the last 1,000 years (MCA, LIA and the twentieth century) and in non-overlapping 50-year bins (Fig. 2c–f) indicates that both the whole twentieth century and the period from 1951 to 2000 are represented by δ^18^O-shell values that are significantly lower than any other period over the last 1,000 years (*P*<0.001; for full *T*-test results, see Supplementary Table 1).

### Calibration against instrumental time series

**Figure 3:**
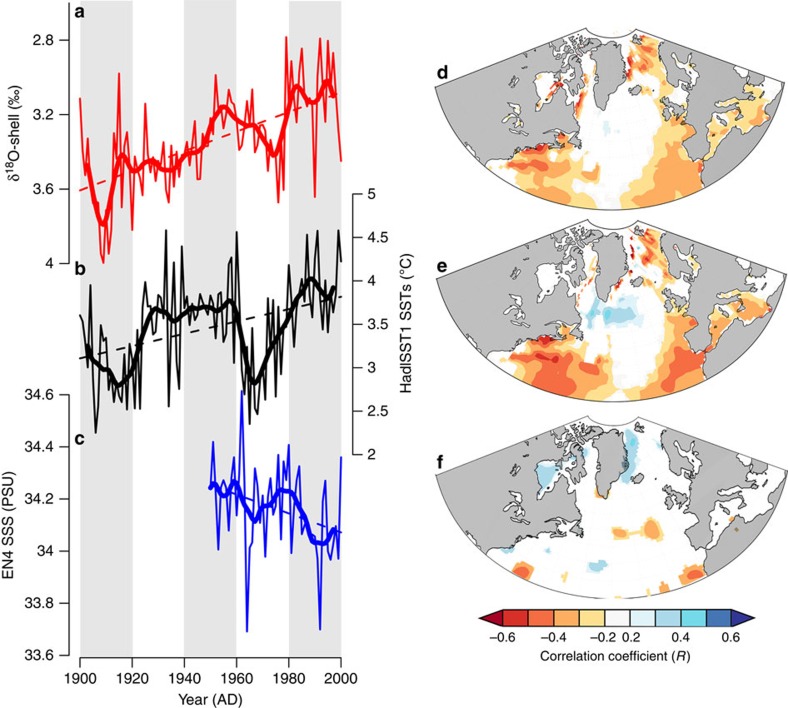
Spatial and temporal examination of the δ^18^O-shell data with instrumental oceanographic time series. (**a**–**c**) Plots of the annual (thin lines) and 10-year low-pass-filtered (thick lines) (**a**) δ^18^O-shell, (**b**) HadISST1 gridded SST data and (**c**) EN4 gridded SSS. (**d**,**e**) Spatial correlations between the δ^18^O-shell and HadISST1 SST over (**d**) the growing season (March to October) and (**e**) the preceding winter (December to February). The correlations are calculated over the period 1870–2000. (**f**) Spatial correlations between the δ^18^O-shell and the EN4 SSS gridded SSS data set. Correlations calculated over the period 1950–2000. All correlations shown are significant with a *P*<0.1. Instrumental displayed in **b**,**c** are derived from a 10° by 20° grid box (65–75°N 10–30°W) north of Iceland. Spatial correlations generated using the KNMI climate explorer (https://climexp.knmi.nl/).

The δ^18^O-shell data were compared with modern temporal and spatial oceanographic instrumental data, to determine the dominant environmental driver of variability (SWT or salinity) and its geographical extent (Fig. 3). The temporal and spatial correlation analyses identified significant correlations between the δ^18^O-shell record and SST (annually resolved correlation *r*=−0.26, *P*<0.1, calculation period 1900–2000) and sea surface salinity (SSS; annually resolved correlation *r*=0.43; *P*<0.1, calculation period 1950–2000). The spatial correlation analyses show that the δ^18^O-shell record correlates significantly (*P*<0.01) with mean growing season (April to October) SSTs over much of the Greenland, Iceland and Norwegian Sea, whereas weaker, yet still significant (*P*<0.1), correlations are also identified over wider regions of the North Atlantic incorporating portions of the Gulf Stream/North Atlantic Current trajectory. Analysis of the δ^18^O-shell record with the preceding winter (December to February) SSTs strengthens the spatial correlation across the wider regions of the North Atlantic. These spatial correlations also indicate a significant positive correlation between sub-polar gyre SST and the δ^18^O-shell record over the preceding winter months (Fig. 3e). The increased strength of these correlations probably reflects the propagation time for variability in these portions of the North Atlantic to influence the waters transported by the NIIC at the sampling site. In addition, although the spatial correlation analyses indicate a significant correlation between the δ^18^O-shell record and SSS on the North Icelandic shelf, the absence of a significant spatial correlation between the δ^18^O-shell data and SST in this region is likely to be associated with differences between SST and bottom (∼80 m water depth) SWTs recorded by *A. islandica*.

Temporal examination of the correlation coefficients identified using the spatial correlation analyses indicates that SST variability on the North Icelandic shelf contains a weakly significant correlation with the annually resolved δ^18^O-shell data (*r*=−0.26, *P*<0.1). However, the SST data explain 36% of the variance (*P*<0.05) in the 10-year loess first-order low-pass-filtered δ^18^O-shell record over the twentieth century and 55% of the variance over the period from 1950 to 2000. Examination of the correlations calculated using linear detrended data indicates that the δ^18^O-shell data contains reduced skill at reconstructing inter-annual variability (linear detrended annually resolved *r*=−0.04, *P*>0.1), but is sensitive to decadal scale SST variability over the twentieth century (linear detrended 10-year low-pass-filtered *r*=−0.37, *P*<0.1). Owing to a lack of instrumental data, it is not possible to evaluate whether the reduction in skill is due to a lack of sensitivity to SSTs or due to differences between SST and bottom water temperatures. Linear regression analysis of the δ^18^O-shell record and North Icelandic shelf SSS data indicates a significant positive correlation with the annually resolved data over the period from 1950 to 2000 (*r*=0.43, *P*<0.01), suggesting that salinity variability explains 18% of the annually resolved variability in the δ^18^O-shell record. Examination of correlations calculated using linear detrended annually resolved and 10-year loess first-order data indicate consistent correlations between the δ^18^O-shell record and SSS data, suggesting that the δ^18^O-shell record is likely to be sensitive to both inter-annual and longer-term SSS variability (annually resolved *r*=0.43, *P*<0.01; 10-year low-pass-filtered *r*=0.46, *P*=0.25; linear detrended annually resolved *r*=0.38, *P*<0.05; linear detrended 10-year low-pass-filtered *r*=0.23, *P*=0.6; all correlations calculated over the period from 1950 to 2000). Further analyses of the coherence between the δ^18^O-shell record and combined SST and SSS variability using multiple linear regression indicate that the combined SST and SSS variability can explain 19% of the variance in the annually resolved and 63% of the variance in the decadal δ^18^O-shell variability over the period 1950–2000 (*P*<0.01). The level of correlation identified using the multiple regression analyses is stable using the linear detrended SSS and SST data (*r*=0.40 and 0.78 for annually resolved and decadal smoothed linear detrended data, respectively), indicating the δ^18^O-shell record is sensitive to both inter-annual and multi-decadal scale variability in combined SSTs and SSSs. We therefore consider our δ^18^O-shell series to qualitatively represent seawater density on the North Icelandic shelf, which is the combined product of SWT and salinity^30^.

## Discussion

Linear regression indicates a weakly significant correlation between variability in our δ^18^O-shell record and the North Icelandic shelf regional marine radiocarbon reservoir ages (Δ*R*)^21^ (*r*=−0.39±0.29, *P*=0.056; Fig. 2 and Supplementary Figs 4 and 5). The Δ*R* data represent the regional difference from the global mean ocean radiocarbon reservoir age, which on the North Icelandic shelf also reflects the degree of entrainment of relatively old (with respect to carbon) polar AIW into the younger SPMW transported by the NIIC^21^,^25^. The coherence between the North Icelandic shelf Δ*R*^21^ and δ^18^O-shell series indicates that during periods characterized by relatively older (younger) Δ*R* values, due to a relative increase (decrease) in the proportion of AIW within the NIIC, the δ^18^O composition of the shell aragonite increases (decreases), indicative of an increase (decrease) in the density of the ambient seawater bathing the site.

**Figure 4:**
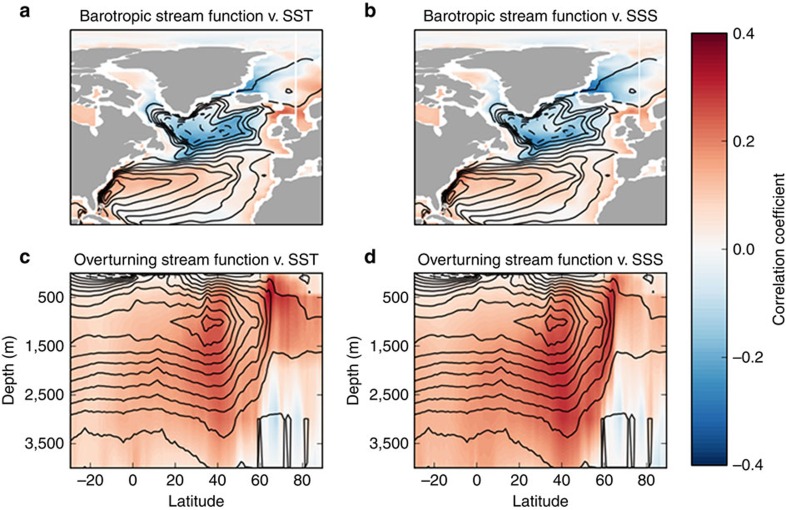
Model analyses of the coherence between North Icelandic SST and SSS variability and North Atlantic circulation. Correlation between annual North Icelandic SSTs against (**a**) barotropic stream function and (**c**) overturning stream function, and SSS against (**b**) barotropic stream function and (**d**) overturning stream function, in the North Atlantic derived from an ensemble CMIP5 preindustrial control simulation. The solid black lines show the time-mean stream functions.

It has previously been proposed that changes in the strength of the Atlantic Meridional Overturning Circulation are linked to variability in the degree of AIW entrainment into the NIIC and therefore the SPMW/AIW composition of the waters influencing the North Icelandic shelf^21^,^31^,^32^. Although instrumental oceanographic observations are currently of insufficient length to evaluate this suggestion, evidence derived from an ensemble of 17 Coupled Model Intercomparison Project phase 5 (CMIP5) unforced pre-industrial control simulations suggests that changes in SST and SSS on the North Icelandic shelf correlate significantly with inter-annual and multi-decadal scale variability in North Atlantic barotropic and overturning stream functions (Fig. 4 and Supplementary Fig. 1). The stability of these correlations, irrespective of the smoothing function applied, indicates that the SST and SSS (along with the associated seawater density) variability on the North Icelandic shelf are sensitive to changes in Atlantic circulation dynamics (Supplementary Fig. 1).

To better constrain the uncertainties associated with the role that the ocean system plays within the wider climate system, it is necessary to assess quantitatively the degree to which ocean variability is related to external forcing mechanisms. To do this we compare the new δ^18^O-shell record to millennial climate forcing time series (volcanics, TSI and modelled combined forcings), NHSAT, derived from a composite dendrochronological record^11^, and Greenland air temperatures, derived from an ice core stack^13^.

**Figure 5:**
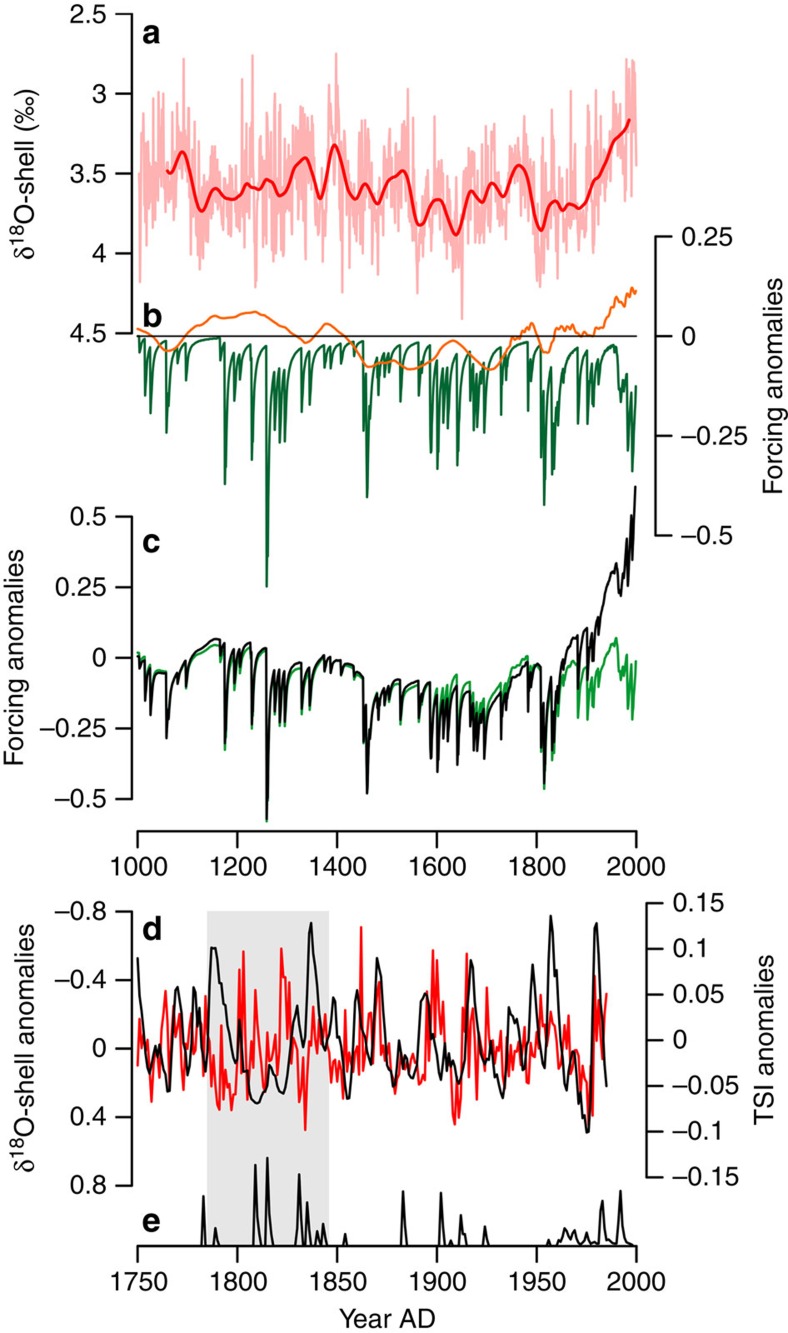
The δ^18^O-shell series and climate forcings. Comparison between (**a**) the inverted δ^18^O-shell anomalies (annually resolved and 50-year loess low-pass-filtered data shown by the pink and red lines, respectively) with (**b**) individual climate forcing indices (TSI and volcanics shown by the orange and green lines, respectively), and (**c**) combined natural forcings (solar and volcanics; green line) and combined total forcing (solar, volcanics, greenhouse gases, aerosols; black line). (**d**) Plot of the 50-year loess first-order high-pass-filtered TSI (black line) and inverted δ^18^O-shell anomalies (red line) over the period AD 1750–1997. (**e**) Timing of volcanic eruptions over the AD 1750–1997 period. The grey box highlights the 2 consecutive 30 periods following the Laki (1783) and Tambora (1815) volcanic eruptions, which probably dominate the climate signal over this period. Climate forcing data from Crowley^2^.

Estimates of past TSI variability can be characterized into two distinct intervals, the observed period (∼AD 1750 to present) and the proxy-generated reconstruction period (years before ∼AD 1750)^2^,^33^. The proxy reconstruction of TSI, derived from radiogenic isotopes (^10^Be and ^14^C) is typically deficient in the high-frequency component of the solar variability spectrum (Supplementary Fig. 6). The observed TSI period however captures both the longer-term trends, such as the gradual increase in TSI since the end of the Maunder Minimum in the early AD 1700s and high-frequency variability such as the 11-year sunspot cycle^2^,^33^. We identify a significant correlation between the δ^18^O-shell series and TSI over the last 1,000 years (annual resolution *r*=0.27, *P*<0.05; decadal resolution *r*=−0.34, *P*<0.05). The correlation is significantly stronger over the observed period of AD 1750–1997 (*r*=−0.68 *P*<0.05), suggesting that TSI variability may explain up to 47% of the variance in seawater density over this period. The coherence spectra of the correlations show that the increase in correlation over the observed period is not likely to be due to low-frequency variability, as similar levels of coherence were identified across these portions of the spectrum for both the observed period and the entire millennium (Supplementary Fig. 6). However, over the observed period, significant correlations (*r*=−0.39, *P*<0.01) were identified within the high-frequency domains (using 50-year loess first-order high-pass-filtered data; Fig. 5d). This high-frequency component of the solar spectrum is unrepresented in the proxy reconstruction derived from radiogenic isotopes (Supplementary Fig. 7). Although these analyses and several previous studies (e.g. refs 16, 17) indicate that solar variability probably plays a role as a natural driver of marine variability, the presence of several large volcanic eruptions over the observed period and the lack of any reliable proxy for high-frequency solar variability over earlier parts of the last millennium complicates the interpretation of the role of solar variability in driving oceanographic variability.

**Figure 6:**
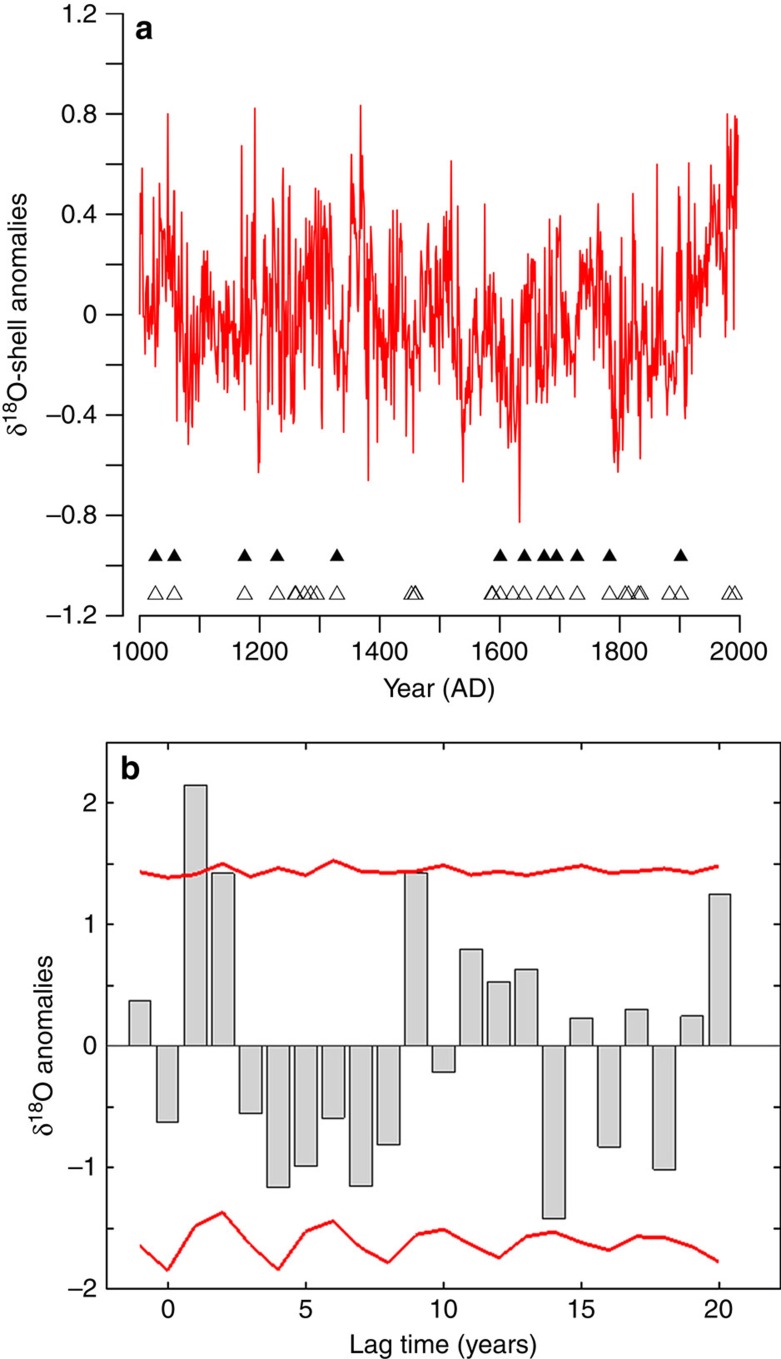
SEA of the δ^18^O-shell series. The SEA was conducted between the 12 largest volcanic eruptions of the last 1,000 years and the annually resolved δ^18^O-shell series. (**a**) The annually resolved δ^18^O-shell series (red line); the triangles indicate the timing of the 12 (black filled triangles) and 30 (open triangles) largest volcanic eruptions, respectively. (**b**) SEA analysis of the δ^18^O-shell series using the 12 largest eruptions. The grey bars indicate the mean δ^18^O-shell series anomalies in over the 12 analysis windows. The red lines indicate the 95% significance level derived using a bootstrapping methodology.

We examined the influence of volcanic aerosols injected into the stratosphere, a consequence of large explosive volcanic eruptions, on the δ^18^O-shell series by conducting a superposed epoch analysis (SEA^34^; Fig. 6). We find a significant positive shift in the δ^18^O-shell anomalies in the first (*P*<0.05) and second (*P*<0.1) year following volcanic eruptions. This result is consistent using two approaches to the SEA (see Supplementary Fig. 7). Although evidence from Swingedouw *et al*.^35^ suggest that volcanic eruptions can have an influence on the North Atlantic that spans several decades, as part of a bi-decadal oscillation, our analyses only indicates a significant response of the marine system in the two years following the largest eruptions of the last 1,000 years. The bi-decadal oscillation present in the SEA analyses of the δ^18^O-shell series, although similar in amplitude and timing to the previously suggested North Atlantic circulation variability associated with volcanic forcing, is not statistically significant^35^. These results indicate that seawater densities north of Iceland show a rapid response to volcanic forcing with an immediate decrease in seawater density over the two years following the eruption. The significant response of the δ^18^O-shell series to the volcanic forcings probably explains the drop in correlation observed between the δ^18^O-shell series and TSI over the period from 1783 to 1843. This period is characterized by three large eruptions, Laki in 1783, an unknown eruption in 1809 and Tambora in 1815, which probably drive the climate signal over this interval.

These analyses indicate that both the TSI and volcanic aerosol forcings are playing a major role as natural drivers of high-frequency (inter-annual to multi-decadal) marine variability on the North Icelandic shelf. However, the comparison between the δ^18^O-shell record and the combined climate forcing index (using TSI, volcanics, stratospheric aerosols and greenhouse gases; Fig. 5c and ref. 2) suggests that, even with the gradual 0.01% increase in TSI since the Maunder Minimum^3^, natural climate forcings alone cannot account for the significant change in δ^18^O-shell values over the industrial period.

**Figure 7:**
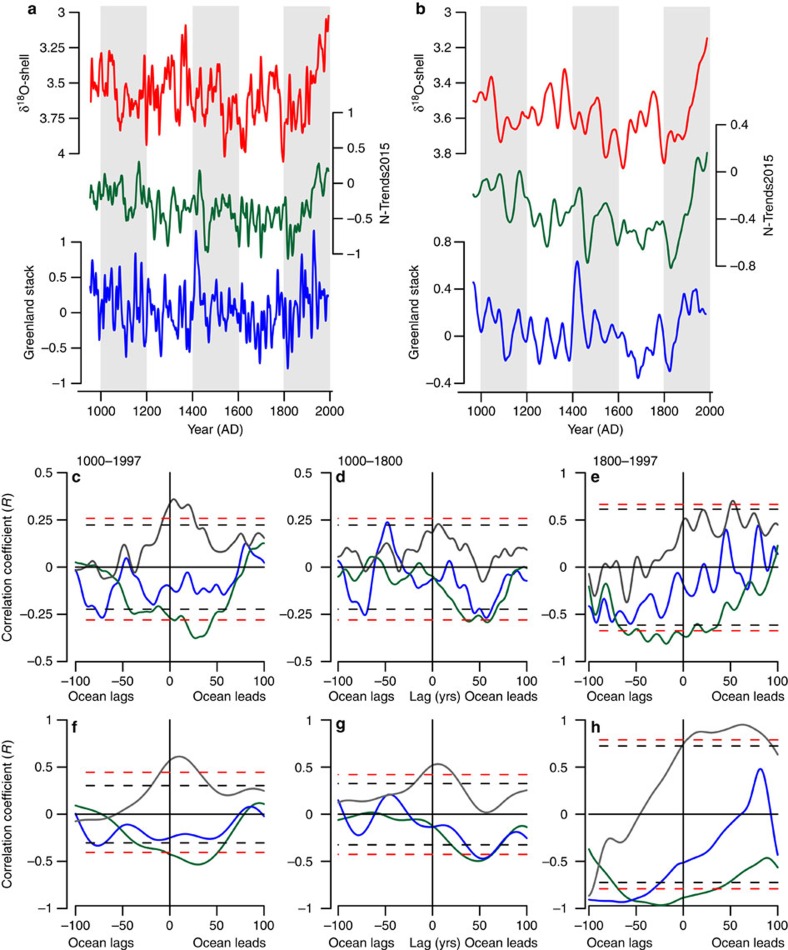
Lead-lag correlation analysis between the inverted δ^18^O-shell anomalies and NHSATs. NHSATs derived from a composite dendrochronological series^11^ and Greenland air temperatures derived from a stack of Greenland ice cores^13^. (**a**) Ten-year and (**b**) 50-year loess first-order low-pass-filtered δ^18^O-shell anomalies (red lines), Northern Hemisphere air temperatures (green lines) and Greenland air temperatures (blue lines). (**c**–**h**) Lead–lag correlation plots calculated between the δ^18^O-shell series and Northern Hemisphere air temperatures (green lines), the δ^18^O-shell series and Greenland air temperatures (blue lines), and Northern Hemisphere air temperatures and Greenland air temperatures (grey lines). The correlations are calculated over three periods. (**c**,**f**) Correlations calculated over the entire millennium. (**d**,**g**) Correlations calculated over the pre-industrial period (AD 1000–1800). (**e**,**h**) Correlations calculated over the industrial period (AD 1800–1997). Data used in the correlations in plots **c**–**e** were 10-year loess first-order low-pass filtered, whereas the data used to calculate the correlations in plots **f**–**h** were 50-year loess first-order low-pass filtered. The dashed black and red lines in plots (**c**–**h**) represent the respective 90 and 95% significance levels calculated using 1,000 Monte Carlo simulations using the Ebisuzaki method^37^.

Given the precisely dated nature of the δ^18^O-shell series and its sensitivity to sub-polar North Atlantic variability, these data present the first opportunity to assess quantitatively the previously untestable hypothesis that ocean circulation dynamics might have played an active role in driving the key climate transitions of the last 1,000 years. We have therefore examined the timing of variability in the absolutely dated δ^18^O-shell series relative to reconstruction of NHSAT, derived from a composite dendrochronological series^11^ and individual tree ring series, and Greenland air temperatures, derived from an ice core stack^13^, over the last 1,000 years (Fig. 7 and Supplementary Figs 9 and 10). Lead–lag correlation analysis, conducted between the normalized δ^18^O-shell anomalies and NHSAT and Greenland air temperatures, indicates that over the pre-industrial period (AD 953–1800) multi-decadal to centennial frequency atmospheric temperature variability lagged changes in the δ^18^O-shell seawater density anomalies by 40±30 years (peak correlation *r*=−0.27 and −0.29, *P*<0.05 against NHSAT and Greenland air temperatures, respectively). Such a temporal offset is beyond the dating uncertainty of these records. The lead of North Icelandic shelf hydrographic variability over both the NHSAT and Greenland air temperature series strongly indicates that ocean variability played an active role in driving the major pre-industrial climate variability of the past 1,000 years. The coincident variability observed in the Greenland air temperature and wider NHSAT records argues against the possibility that the ocean lead we observe is driven by regional changes in atmospheric circulation patterns.

The results of the lead–lag analysis of the reconstructions are further validated using the available ensemble of CMIP5 unforced pre-industrial control simulations (Supplementary Fig. 12). The CMIP5 preindustrial control simulations contain no external forcing; thus, all climate variability is internally generated^9^. Lead–lag analysis of the modelled North Icelandic shelf seawater density and NHSAT show that, consistent with the proxy records, the ocean variability leads the atmosphere by 10±10 years (Supplementary Fig. 12)^18^,^19^,^35^. The offset in the magnitude of ocean–atmosphere lag between model and real-world data are likely to be a result of differences in the detail of the surface and meridional circulations between models, and between the models and reality.

In comparison with the preindustrial interval, the modern trends (AD 1800–2000) in the NHSAT records relative to the δ^18^O-shell anomalies, coupled with the lead–lag analyses, suggests a reversal of the pre-industrial ocean–atmosphere coupling over the industrial period (AD 1800–2000) with changes in ocean dynamics more closely coupled in time, or lagging, increases in atmospheric surface air temperatures (Fig. 7). The close coupling identified by the proxy records over the industrial period is in agreement with the timing of coupling observed between instrumental NHSAT and sub-polar North Atlantic SSTs over the period AD 1880–2015 (Supplementary Fig. 11). The shift in coupling between the pre-industrial and industrial time periods is likely to be driven by a change in the balance between top-down and bottom-up forcings^19^.

The lead–lag correlations, coupled with the correlation of the δ^18^O-shell data with volcanic and high-frequency TSI forcing, suggest that at higher frequencies (<20 years) the ocean–atmosphere systems is closely coupled with periodic volcanic eruptions and high-frequency solar variability (for example, the sun spot cycle) driving a rapid response in both the atmosphere and ocean systems^35^. The lead–lag analyses suggest that at lower frequency domains (multi-decadal to centennial) marine variability is playing an active role in driving atmospheric variability. Such a divergence in the lagged response of the ocean and atmosphere systems at different frequency domains is detectable in the modern instrumental observations. Examination of a suite of instrumental SST and NHSAT's indicates that over the twentieth century the atmosphere leads the ocean system at lower frequencies (multi-decadal; see Supplementary Fig. 11), probably driven by the faster response of the atmosphere to the warming influence of greenhouse gases. However, examination of the high-frequency variability (20-year high-pass-filtered data; Supplementary Fig. 11) implies a tighter coupling with high-frequency forcings/feedback mechanisms causing synchronous variability across both the ocean and atmosphere systems. These data suggest that during the pre-industrial period internal variability and feedback mechanisms within the North Atlantic substantially mediate the response of the climate system to top–down forcing (TSI, volcanic, atmospheric aerosols and greenhouse gases). Although, over the industrial period these data imply that internal oceanic mediation of top–down forcing has been overcome by the rate and nature of the NHSAT increase forced by increasing greenhouse gas concentrations.

These findings have implications for the interpretation of the modern climate system, as they highlight the problems that can result from using short modern oceanographic instrumental observations as a representative baseline of natural climate variability. Such records, although capturing a component of natural variability, are probably dominated by the anthropogenic signal. Furthermore, our data demonstrate shortcomings in methodologies that attempt to extend the modern instrumental ocean observations using absolutely dated records derived from terrestrial proxy networks (for example, see ref. 36). With the reconstructed reversal in the timing of ocean–atmosphere coupling, these results question the ability of terrestrial archives alone to reconstruct ocean variability and highlight the need to use independent absolutely dated marine archives to characterize the role ocean dynamics play in the global climate system.

The δ^18^O-shell record presented here provides the first precisely dated annually resolved record of past marine variability that spans the entire last 1,000 years. It provides a long-term baseline for the state of the North Atlantic coupled climate system and demonstrates that the role played by the ocean in naturally forced climate variability should be a key focus if we are to realise the societally crucial step forward in near-term climate prediction.

## Methods

### Shell collection and age model

Live and dead *A. islandica* shell material was collected from 80 m water depth, from the North Iceland shelf (Grimsey, 66° 31.59′ N, 18° 11.74′ W); full details of the shell collection are provided in (ref. 25). An absolutely dated master shell chronology was constructed by cross-dating the live and dead collected shell material using techniques derived from dendrochronology. The statistical cross-dated ages were validated using accelerator mass spectrometry radiocarbon dating. Full details of the construction of the North Iceland shelf growth increment width chronology are available in refs 21, 25, 28. Aragonite calcium carbonate samples were micro-milled from known age *A. islandica* growth increments using a Merchantek New Wave Micro-mill at annual resolution over the period AD 953–2000 and at sub-annual resolution over periods of the twentieth century. All temporal correlations reported were calculated using the Ebisuzaki Monte Carlo method to take into account auto-correlation^37^.

### Stable isotope analyses

Stable isotope measurements were completed using a Thermo Finnigan MAT 252 and 253 isotope ratio mass spectrometers coupled to a Kiel carbonate preparation device at Cardiff University. The isotopic results are reported as a per mil deviation from the Vienna Pee Dee Belemnite scale with an external reproducibility of carbonate standards (NBS 19) better than 0.08‰ for δ^18^O.

### Construction of the δ^18^O-shell series

The δ^18^O-shell series was constructed from the analysis of 1492 annually resolved aragonite samples. These samples originate from 21 individual shells that were previously cross-dated. Replicates were taken at each overlap between shells. Further replication was conducted over the twentieth century where replication in the chronology was strongest. The arithmetic mean was calculated in years containing multiple δ^18^O analyses, to construct a single series with one value per year. Supplementary Fig. 2 highlights all the years with replicated δ^18^O analyses. Contrary to the development of growth increment width chronologies, the δ^18^O-shell series requires no application of detrending methodologies as the δ^18^O-shell series contains no ontogenetic trends.

### Sub-annually resolved δ^18^O analyses

Sub-annually (sub-incremental) resolved δ^18^O-shell samples were analysed from shells covering the latter half of the twentieth century, to determine the seasonality of growth and therefore to determine the seasonal representation of the annually resolved growth increment samples. The sub-annual δ^18^O-shell data were converted to SWTs using modified Grossman and Ku^38^ aragonite palaeotemperature equation^39^. The ambient water δ^18^O values (δ^18^O_w_) were derived from seawater salinity timeseries using the North Iceland salinity mixing line equation^40^. These sub-annually resolved reconstructed δ^18^O-shell SWT were then compared with seasonal records of local SWT derived from the Grimsey oceanographic instrumental timeseries (Supplementary Fig. 3). Comparison of the observed and reconstructed SWT allowed the determination of the period of growth and the timing of the formation of the growth check. The sub-annually resolved reconstructed δ^18^O-shell SWT contains a range of temperatures that are coherent with SWT during the period of March through to October (Supplementary Fig. 3). Comparison of the relative drilling position within each growth increment (calculated as the percentage of total annual growth) against the corresponding time of year represented by the corresponding sub-annual δ^18^O-shell sample provides an estimation of the seasonal growth rate and therefore provides an estimate of the seasonal weighting of the annually resolved δ^18^O-shell samples (Supplementary Fig. 3C). These analyses suggest that although the majority of growth takes place during the period of June–October, the full growth season extends from March through to October. These results are in agreement with previously published records of *A. islandica* growth from Iceland^41^.

### Superposed epoch analysis

The SEA approach aligns windows of the δ^18^O-shell record based on the timing of the selected volcanic eruptions and calculates an average of the time series. The averaging of these windows removes the relative influence of non-aligned climate forcing from the record increasing the signal to noise ratio allowing for the identification of a volcanic induced climate signal if present^34^. A Monte Carlo approach was used to assess the statistical significance of the identified climate signal. We performed the SEA using two approaches and compared the results. For the first approach we selected the 30 years with the highest volcanic aerosol concentrations, which probably represent the largest 30 volcanic eruptions of the last 1,000 years from the Crowley^2^ volcanic index. In this approach, all 30 eruptions were used regardless of whether other large eruptions occurred during the analysis interval. In the second approach we initially selected the same 30 volcanic eruptions as in the first approach, but discarded any eruption that was followed by another large eruption within the analysis window (following 20 years) similar to approaches taken by Swingedouw *et al*.^35^. This filtering process led to the analysis of 12 volcanic eruptions.

### Lead–lag analysis of Northern Hemisphere SAT reconstructions

Pearson's correlation coefficients were calculated over the pre-industrial (AD 1000–1800) and industrial (AD 1800–2000) period windows with lead–lags of ±100 years (1 year incremental steps). In total, 13 individual tree-ring width and multi-proxy network derived NHSAT records were used in the analysis. The raw δ^18^O-shell anomalies and NHSAT records were filtered using a 10-year loess first-order low-pass filter, to equalize the highest frequency variability across the records, and 50-year loess first-order low-pass filter, to examine the low-frequency components of variability. The lead–lag correlations were calculated using the 10-year and 50-year low-pass-filtered data, respectively. Significance levels for the correlations were determined using the Ebisuzaki method^37^.

Examination of the lead–lag analyses using an increasing smoothing function (running mean smoothing; Supplementary Fig. 10) highlights that over the pre-industrial period there is a significant correlation between the NHSATs and the δ^18^O-shell data with a lag of ∼50 years. However, the lead–lag correlations of the entire period show little lag. Examination of the running lead–lag correlation analysis indicates that the 50-year lagged correlation is persistent over the period AD 1200–1800 with a switch occurring over the industrial period to a negligible lag over the nineteenth and twentieth centuries. The running lead–lag correlations calculated between the 10-year low-pass-filtered data indicate that there is some coherence at around zero year lag; however, there is also a strong correlation at 50 years. This suggests that there is a component of variability that is tightly coupled between the ocean and atmosphere systems.

### Examination of trends in the instrumental record

The ocean–atmosphere system over the modern instrumental record generally shows close temporal coupling; however, subtle differences are present at specific frequency domains. Supplementary Fig. 11 explores the timing of variability and multi-decadal (20-year low-pass-filtered data) and at high frequencies (20-year high-pass-filtered data). The low-pass-filtered data clearly show that the onset of the early- and mid-twentieth century (∼1910 and ∼1960) warming phases in the atmosphere precede the warming in the ocean by around a decade. However, over the same intervals the ocean and atmosphere system shows a close coupling in the high-frequency domain with the inter-annual temperature anomalies remaining coherent over the entire record (Supplementary Fig. 11). These data therefore support the results from the proxy analyses that the temporal alignment of the ocean–atmosphere coupling can differ across different temporal frequency domains.

### CMIP5 pre-industrial control simulation analysis

SST, SSS and surface air temperature data were extracted from the available CMIP5 full pre-industrial control simulations (Supplementary Note 1). The selected model fields were re-gridded onto a 180 × 360° grid using bilinear interpretation. Mean SST and SSS data, calculated within each model across the North Iceland region (25.0°W to 10°W, 65°N to 70°N), were used to calculate North Iceland seawater densities using the UNESCO 1983 algorithm^42^. These data were compared with the model-derived mean NHSAT data using the same lead–lag methodology that was applied to the δ^18^O-shell and dendrochronological air temperature proxy series (Supplementary Fig. 12).

### Data availability

Data are available through the NOAA climate data centre (https://www.ncdc.noaa.gov/paleo/study/20448)

## Additional information

**How to cite this article:** Reynolds, D. J. *et al*. Annually resolved North Atlantic marine climate over the last millennium. *Nat. Commun.* **7,** 13502 doi: 10.1038/ncomms13502 (2016).

**Publisher's note**: Springer Nature remains neutral with regard to jurisdictional claims in published maps and institutional affiliations.

## Supplementary Material

Supplementary Information Supplementary Figures 1-12, Supplementary Table 1, Supplementary Note 1 and Supplementary References

## Figures and Tables

**Figure 1:**
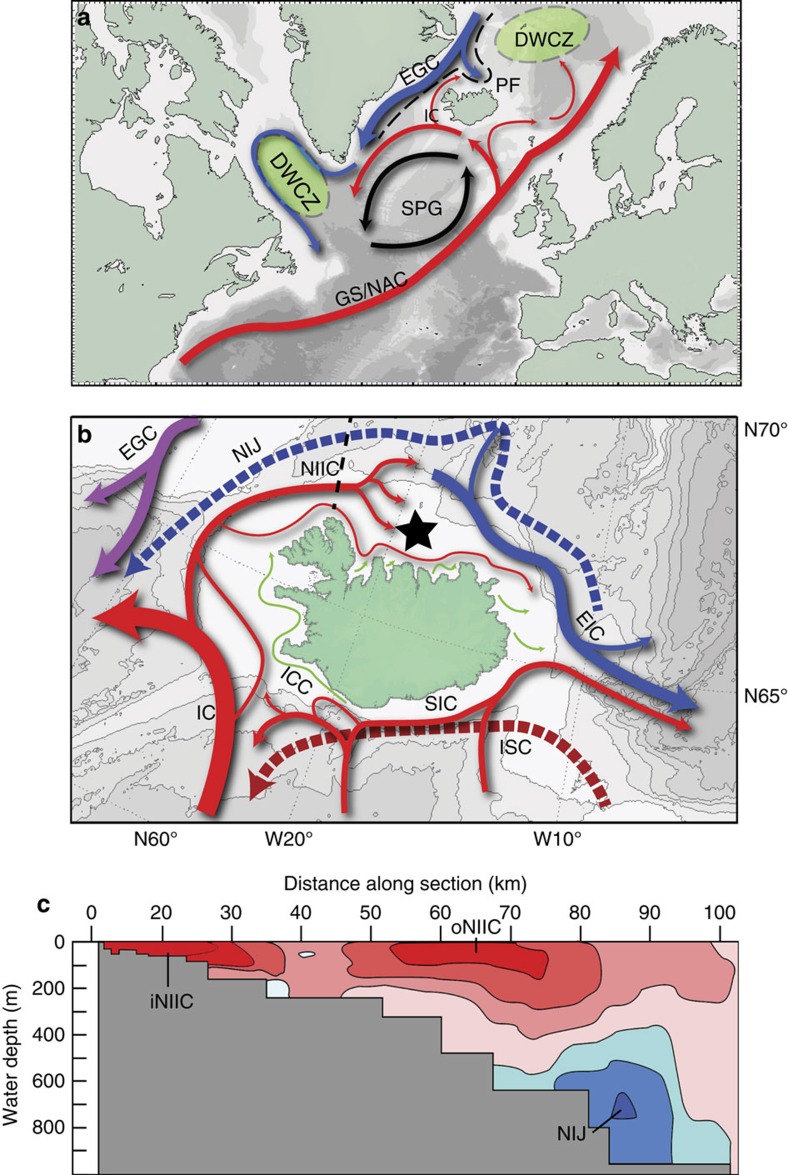
Maps of the modern surface currents of the North Atlantic and Icelandic shelf. (**a**) North Atlantic circulation and (**b**) North Icelandic shelf circulation pattern. Arrows shown in blue correspond to cool and relatively fresh Arctic-sourced waters; arrows shown in red are warm and saline Atlantic-sourced waters; dashed lines correspond to deep currents whilst the solid arrows denote surface currents. The dashed black line in **a** refers to the approximate position of the North Atlantic Polar Front. The dashed black line in **b** refers to the depth transect shown in **c**. The star denotes the location of the shell sampling site (80 m water depth 66° 31.59′ N, 18° 11.74′ W). (**c**) Depth transect across the North Icelandic shelf indicating the direction of flow of each of three dominant water currents at this locality. Red colours indicate an easterly flow whilst blue indicates a westerly flow. DWCZ, deep water convection zones; EGC, East Greenland Current; EIC, East Icelandic Current; GS/NAC, Gulf Stream/North Atlantic Current; IC, Irminger Current; iNIIC, inner NIIC; ISC, Icelandic slope current; NIIC, North Icelandic Irminger Current; NIJ, North Icelandic Jet; oNIIC, outer NIIC; PF, polar front; SIC, South Icelandic Current. (**b**,**c**) Adapted from ref. 27 and the base map from ref. 43.
