# Supplementary material for: Annually resolved North Atlantic marine climate over the last millennium
Source: Nat Commun. 2016 Dec 6;7:13502. doi: 10.1038/ncomms13502 (PMC5150573; doi:10.1038/ncomms13502)
Supplement: Supplementary Information — Supplementary Figures 1-12, Supplementary Table 1, Supplementary Note 1 and Supplementary References [file ncomms13502-s1.pdf]

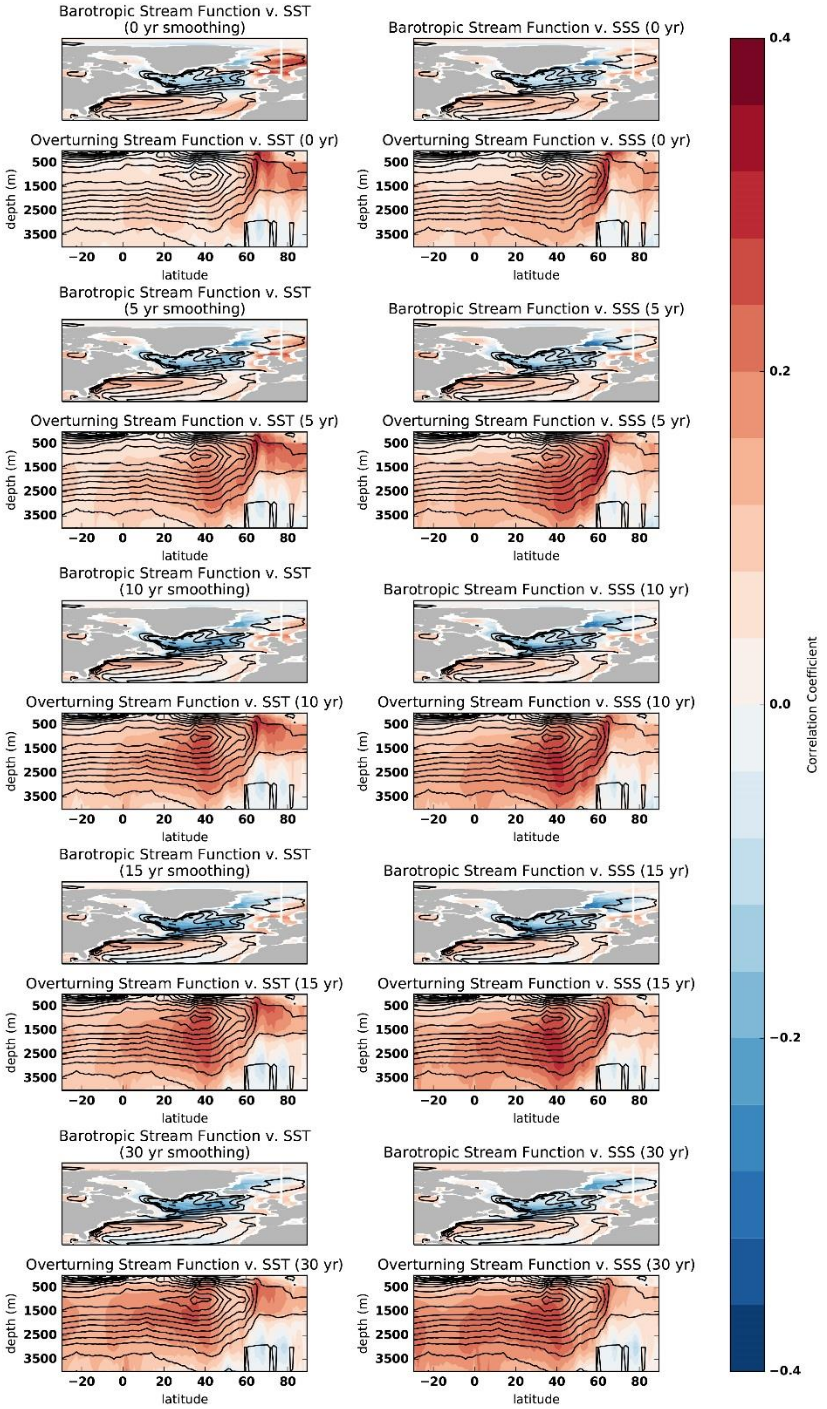

Supplementary Figure 1: Correlation between North Icelandic SSTs and SSS against overturning stream function and barotropic stream function in the North Atlantic derived from an ensemble CMIP5 preindustrial control simulation. The solid black lines show the time-mean stream functions. The correlations are been calculated using a range of low pass filtered data (annual data [zero year smoothing] 5 year, 10 year, 15 year and 30 year low pass filter).

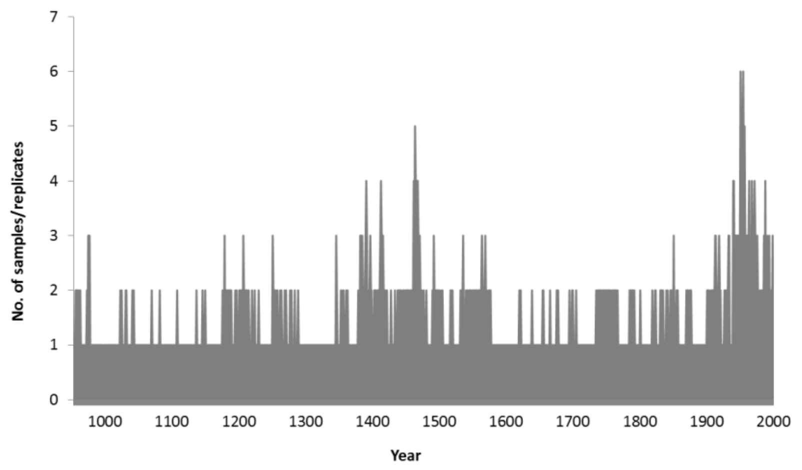

Supplementary Figure 2: Plot indicating the number of annually resolved aragonite samples analysed in the construction of the δ^{18}O-shell series.

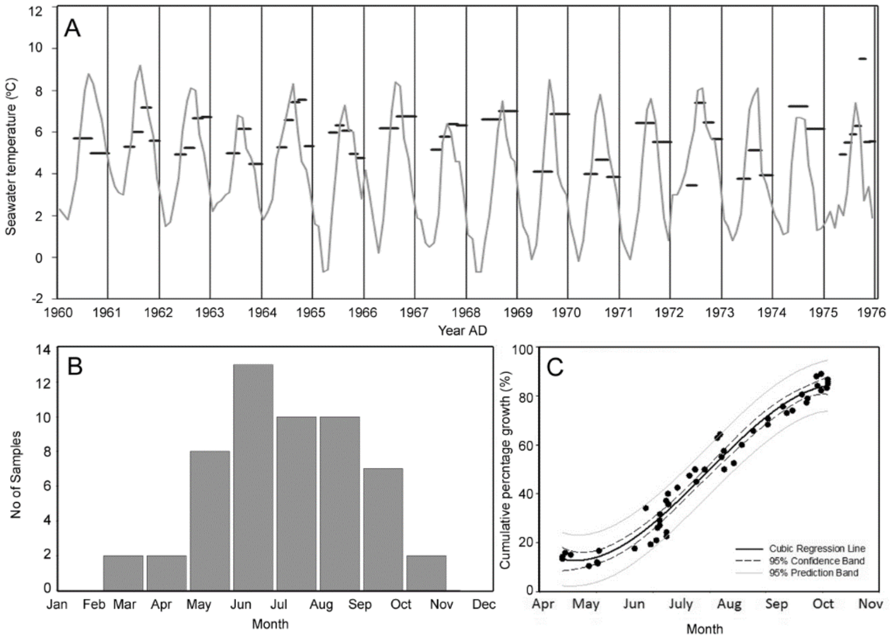

Supplementary Figure 3: A) Sub-annual δ^{18}O-shell SWT data (black bars), derived from 16 *A. islandica* growth increments, plotted against the corresponding monthly seawater temperatures (grey line) from Grimsey NIS. B) Frequency histogram displaying the number of sub-annual samples with reconstructed temperatures that correspond to the respective month. C) Percentage of annual growth completed, measured as the sampling (micromilling) position within the growth increment relative to the overall width of the growth increment, to the modelled time of year with which the δ^{18}O-shell SWT data corresponds, fitted with a polynomial curve with 95% CIs. The gradient of the polynomial curve can be interpreted broadly as the seasonal growth rate.

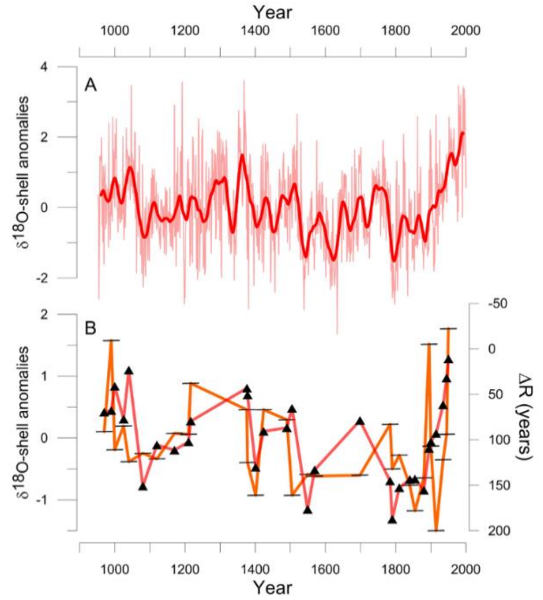

Supplementary Figure 4: A) Annually resolved δ^{18}O-shell anomalies (pale red line) plotted on an inverted anomaly scale fitted with a 30 year first order loess low-pass filter (thick red line). B) North Iceland ΔR[1] (orange line with black bars) plotted with the corresponding values from the 50 year first order loess low pass filtered δ^{18}O-shell anomalies (red line with black triangles).

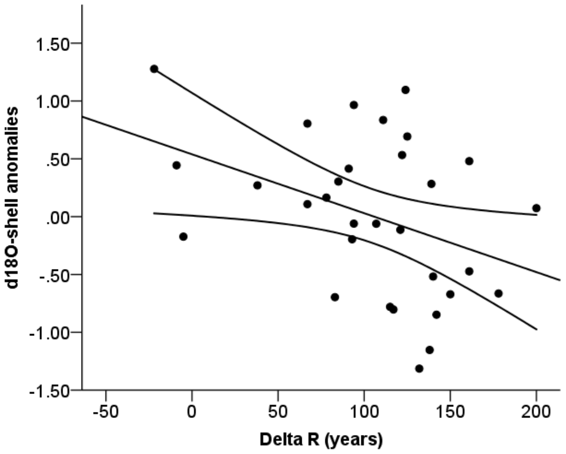

Supplementary Figure 5: Linear regression analysis between the δ^{18}O-shell series and the North Iceland ΔR series[1]. The δ^{18}O-shell data were filtered to match the approximate resolution of the ΔR series (50 year first order loess low pass filter) and only the years with corresponding ΔR data were used in the analyses. R=-0.39±0.29 (95% CI) n=31 P=0.056.

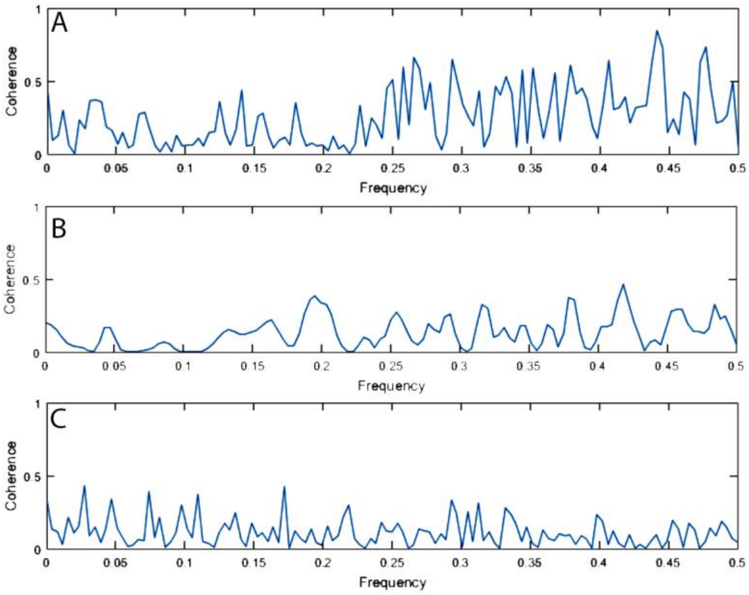

Supplementary Figure 6: Spectral coherence between the $\delta^{18}O$-shell series and total solar irradiance[4] calculated over A) the entire millennium; B) AD 1750-2000; and c) AD 1000-1750.

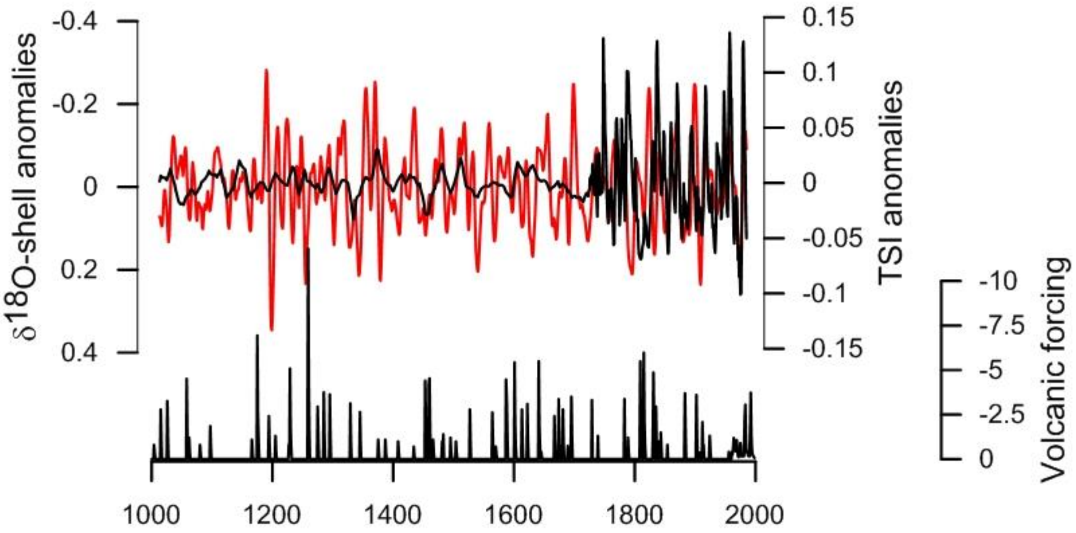

Supplementary Figure 7: Plot of the 50 year high pass filtered δ^{18}O-shell (red line), TSI data (black line) and volcanic (black line in lower panel) forcing timeseries[4] over the last millennium. The TSI data show a marked increase in the amplitude of variability over the period from ca. AD 1750-2000 relative to that over the period AD 1000-1750.

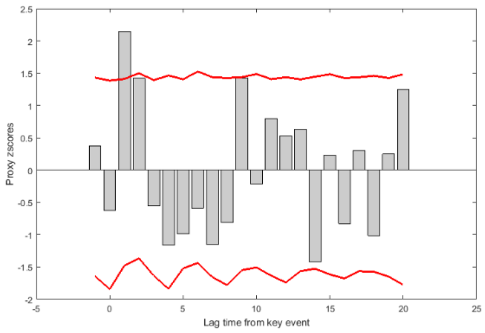

A

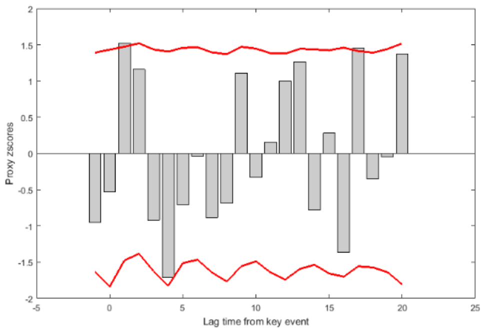

B

Supplementary Figure 8: SEA of the δ^{18}O-shell series using a) the 12, and b) the 30 largest volcanic eruptions of the last millennium. The grey bars represent the mean δ^{18}O-index over 22 year analysis window. The red lines indicate the 95% bootstrapped significance level.

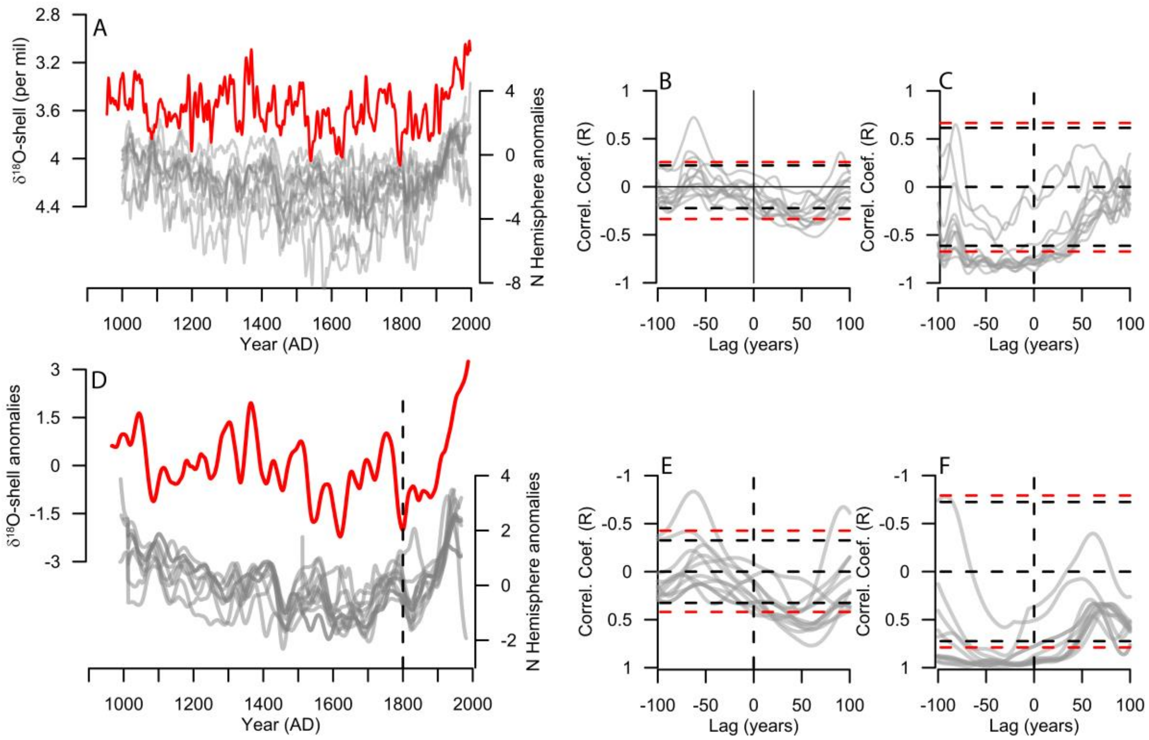

Supplementary Figure 9: Lead lag correlations calculated between the δ^{18}O-shell series and thirteen independent northern hemisphere surface air temperature (NHSAT) reconstructions derived from dendrochronological networks[5-17]. a) Decadal low pass filtered δ^{18}O-shell (red line) and NHSAT reconstructions (grey lines); b and c) lead lag correlations calculated between the decadal smoothed timeseries over the pre-industrial and industrial periods respectively; d) 50-year first order loess δ^{18}O-shell and NHSAT records; e and f) lead lag correlations calculated between the 50 year low pass filtered data over the pre-industrial and industrial periods respectively. b, c, e and f) red and black dashed lines represent the 90 and 95% significance levels taking into account the serial nature of the timeseries. All of the independent NHSAT reconstructions are available through the NOAA palaeo data portal (www.ncdc.noaa.gov/data-access/paleoclimatology-data/datasets).

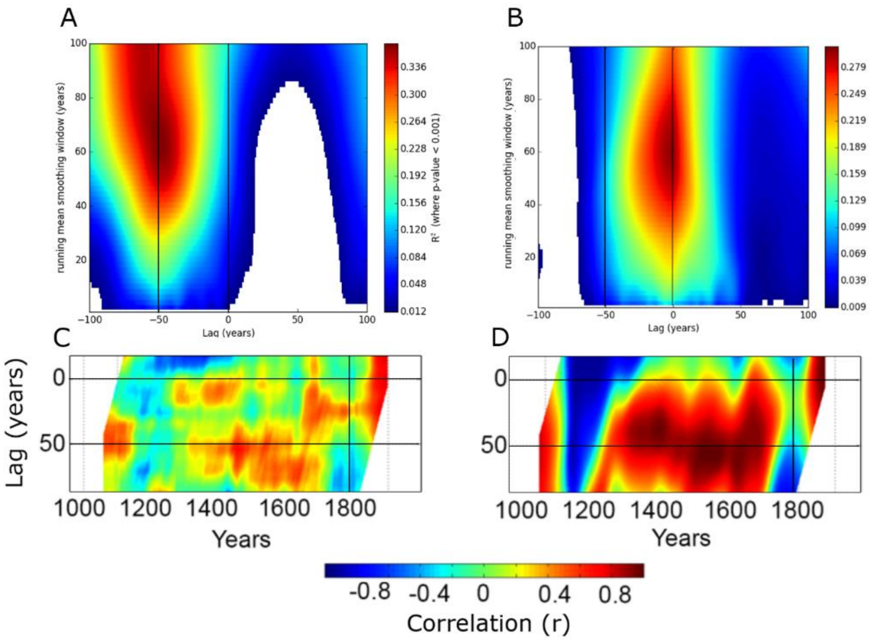

Supplementary Figure 10: Examination of the lead-lag correlations at different frequencies and the stability of the correlations through time. Lead-lag correlation coefficients calculated between the δ^{18}O-shell series and the NHSAT[5] series calculated over A) the pre-industrial period and B) over the entire record using a range of low-pass filtered data calculated using arithmetic running mean with an increasing window length between one and 100 years. Correlations shown with a P<0.001. C-D) Running lead-lag correlation analyses calculated over 201 year running window between the δ^{18}O-shell series and the NHSAT series using C) 10-year low pass filtered data and C) 100-year low pass filtered data; and I) 100-year low pass filtered data.

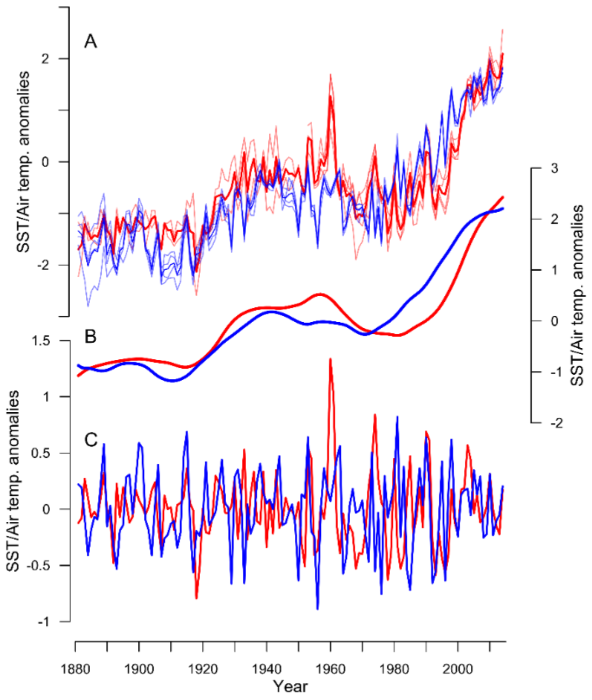

Supplementary Figure 11: A) Normalised Sub-polar North Atlantic SSTs (blue lines) and Northern Hemisphere surface air temperatures (red lines) extracted from the HadISST1[18], ERSST v4[19], HadSST3[20,21], ICOADS[22], GISS250[23], HadCRUT4[24], NCDC V3[25] and Berkeley[26] gridded instrumental datasets. B) 20 year low pass filtered sub-polar North Atlantic SSTs and Northern Hemisphere surface air temperatures (red and blue lines respectively). C) 20 year high pass filtered sub-polar North Atlantic SSTs and Northern Hemisphere surface air temperatures (red and blue lines respectively). The high pass filtered data was calculated by subtracting the 20 year first order loess filtered data (B) from the raw data (A). Instrumental data were extracted from a 10° by 20° grid box (65-75°N 10-30°W) north of Iceland.

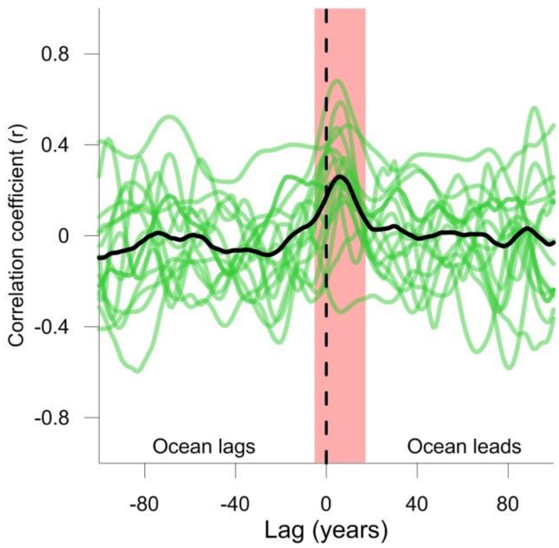

Supplementary Figure 12: Lead-lag correlation analysis between north Iceland seawater density and corresponding Northern Hemisphere surface air temperatures derived from sixteen CMIP5 models (green lines). The black line represents the mean lead-lag correlations of all the models. Shaded red bar indicates period of peak correlation.

Supplementary Table 1: T test statistics comparing A) the 1951-2000 mean δ^{18}O-shell value to the preceding 50 year periods. Means were calculated for 50 year bins with zero years overlap. B) comparing the 20th century with that of the key periods of the last millennium (MCA, LIA, MCA-LIA transition and the entire millennium mean.

A

	Test Value = 1951-2000 mean					
					95% Confidence Interval of the Difference	
Period (AD)	t	df	Sig. (2-tailed)	Mean Difference	Lower	Upper
1901 - 1950	10.708	49	.000	.306	.249	.364
1851 - 1900	15.782	49	.000	.501	.438	.565
1801 - 1850	16.152	49	.000	.512	.448	.576
1751 - 1800	11.323	49	.000	.460	.378	.542
1701- 1750	17.205	49	.000	.388	.342	.433
1651 - 1700	13.112	49	.000	.421	.357	.486
1601 -1650	18.855	49	.000	.611	.546	.676
1551 - 1600	21.174	49	.000	.501	.454	.549
1501 -1550	11.326	49	.000	.457	.376	.538
1451 - 1500	15.217	49	.000	.407	.354	.461
1401 - 1450	14.075	49	.000	.431	.369	.492
1351 - 1400	5.219	49	.000	.214	.132	.297
1301 - 1350	10.684	49	.000	.383	.311	.454
1251 - 1300	9.798	49	.000	.348	.277	.420
1201 - 1250	9.468	49	.000	.353	.278	.428
1151 - 1200	11.093	49	.000	.432	.354	.511
1101 - 1150	20.633	49	.000	.421	.380	.462
1051 - 1100	13.384	49	.000	.462	.393	.532
1001 - 1050	7.705	49	.000	.232	.172	.293
953 - 1000	9.028	47	.000	.308	.239	.376

B

	Test Value = 20th Century Mean				
	t	df	Sig. (2-tailed)	Mean Difference	95% Confidence Interval of the Difference

Period					Lower	Upper
Entire millennium	28.775	1047	.000	.232	.217	.248
LIA	26.938	400	.000	.314	.291	.337
MCA-LIA transition	5.878	98	.000	.164	.108	.219
MCA	17.146	397	.000	.212	.187	.236

Supplementary Note 1:

List of the CMIP5 pre-industrial control simulations, and their references, used to examine the mechanistic relationship between North Iceland sea water density and northern hemisphere air temperatures. CESM1[27], CNRM-CM5[28], CanESM2[29], EC-EARTH[30], GFDL-CM3[31], GFDL-ESM[32], GISS-E2[33], HadGEM2-ES[34], MOHC-HadGEM2-CC[35], IPSL-CM5[36], MIROC-ESM[37], MIROC5[38], MPI-ESM-LR[39], MPI-ESM-MR[39], MRI-CGCM3[40], NorESM1-M[41], NorESM1-ME[42], bcc-csm1-1[43] and inmcm4[44].

Supplementary References

1 Wanamaker, A. D., Jr. *et al.* Surface changes in the North Atlantic meridional overturning circulation during the last millennium. *Nature communications* **3**, 899, doi:10.1038/ncomms1901 (2012).

2 Mann, M. E. *et al.* Global signatures and dynamical origins of the Little Ice Age and Medieval Climate Anomaly. *Science* **326**, 1256-1260, doi:10.1126/science.1177303 (2009).

3 Rahmstorf, S. *et al.* Exceptional twentieth-century slowdown in Atlantic Ocean overturning circulation. *Nature Climate Change*, doi:DOI: 10.1038/NCLIMATE2554 (2015).

4 Crowley, T. J. Causes of Climate Change Over the Past 1000 Years. *Science* **289**, 270-277, doi:10.1126/science.289.5477.270 (2000).

5 Wilson, R. *et al.* Last millennium northern hemisphere summer temperatures from tree rings: Part I: The long term context. *Quaternary Science Reviews* **134** 1-18, doi:10.1016/j.quascirev.2015.12.005 (2016).

6 Moberg, A., Sonechkin, D.M. Holmgren, K. Datsenko N.M. & Karln W. Highly variable Northern Hemisphere temperatures reconstructed from low- and high-resolution proxy data. *Nature* **433**, 613-617 (2005)

7 Ammann, C.M. and Wahl, E.R. The importance of the geophysical context in statistical evaluations of climate reconstruction procedures. *Climatic Change* **85**, 71-88 (2007)

8 Briffa K.R., Jones, P.D. Schweingruber F.H., & Osborn, T.J. Influence of volcanic eruptions on Northern Hemisphere summer temperature over the past 600 years. *Nature* **393**, 450-455 (1998)

9 Crowley, T. J. & Lowery, T. S. How warm was the medieval warm period? *Ambio* **29**, 51-54 (2000)

10 D'Arrigo, R., Wilson, R. & Jacoby, G. On the long-term context for late twentieth century warming. *Journal of Geophysical Research* **111**, D03103 (2006)

11 Esper, J., Cook, E.R. & Schweingruber, F.H. Low-Frequency Signals in Long Tree-Ring Chronologies for Reconstructing Past Temperature Variability. *Science* **295**, 22 (2002)

12 Hegerl, G. C., Crowley, T. J., Hyde, W. T. & Frame, D. J. Climate sensitivity constrained by temperature reconstructions over the past seven centuries. *Nature* **440,** 7087, 1029-1032 (2006)

13 Huang, S. Merging Information from Different Resources for New Insights into Climate Change in the Past and Future. *Geophysical Research Letters* **31**, L13205 (2004)

14 Kobashi, T., Goto-Azuma, K. Box, J.E. Gao, C.C. & Nakaegawa, T.Causes of Greenland temperature variability over the past 4000 yr: implications for northern hemispheric temperature changes. *Climate of the Past* **9,** 2299-2317 (2013)

15 Christiansen, B. & Ljungqvist, F.C.The extra-tropical Northern Hemisphere temperature in the last two millennia: reconstructions of low-frequency variability. Climate of the Past 8, 765-786 (2012)

16 Mann, M.E., Bradley, R.S. & Hughes, M.K. Northern Hemisphere temperatures During the Past Millennium: Inferences, Uncertainties, and Limitations. *Geophysical Research Letters* **26**, 759 (1999)

17 Shi, F., Yang, B., Mairesse, A., von Gunten, L., Li, J., Bräuning, A., Yang, F., & Xiao, X. Northern Hemisphere temperature reconstruction during the last millennium using multiple annual proxies. *Climate Research* **56**, 231-244 (2013)

18 Rayner, N. A. *et al.* Global analyses of sea surface temperature, sea ice, and night marine air temperature since the late nineteenth century. *Journal of Geophysical Research-Atmospheres* **108**, doi:10.1029/2002jd002670 (2003).

19 Smith, T. M., Reynolds, R. W., Peterson, T. C. & Lawrimore, J. Improvements to NOAA's historical merged land-ocean surface temperature analysis (1880-2006). *Journal of Climate* **21**, 2283-2296, doi:10.1175/2007jcli2100.1 (2008).

20 Kennedy, J. J., Rayner, N. A., Smith, R. O., Parker, D. E. & Saunby, M. Reassessing biases and other uncertainties in sea surface temperature observations measured in situ

since 1850: 2. Biases and homogenization. *Journal of Geophysical Research-Atmospheres* **116**, doi:10.1029/2010jd015220 (2011).

21 Kennedy, J. J., Rayner, N. A., Smith, R. O., Parker, D. E. & Saunby, M. Reassessing biases and other uncertainties in sea surface temperature observations measured in situ since 1850: 1. Measurement and sampling uncertainties. *Journal of Geophysical Research-Atmospheres* **116**, doi:10.1029/2010jd015218 (2011).

22 Woodruff, S. D., Diaz, H. F., Kent, E. C., ReynoldS, R. W. & Worley, S. J. *The evolving SST record from ICOADS*. Vol. 33 (2008).

23 Hansen, J., Ruedy, R., Sato, M. & Lo, K. GLOBAL SURFACE TEMPERATURE CHANGE. *Reviews of Geophysics* **48**, doi:10.1029/2010rg000345 (2010).

24 Morice, C. P., Kennedy, J. J., Rayner, N. A. & Jones, P. D. Quantifying uncertainties in global and regional temperature change using an ensemble of observational estimates: The HadCRUT4 data set. *Journal of Geophysical Research-Atmospheres* **117**, doi:10.1029/2011jd017187 (2012).

25 Jones, P. D. & Moberg, A. Hemispheric and large-scale surface air temperature variations: An extensive revision and an update to 2001. *Journal of Climate* **16**, 206-223, doi:10.1175/1520-0442(2003)016<0206:halssa>2.0.co;2 (2003).

26 Rohde, R., Muller, R.A., Jacobsen, R., Muller, E., Perlmutter, S., Rosenfeld, A., Wurtele, J., Groom, D., Wickham, C. A New Estimate of the Average Earth Surface Land Temperature Spanning 1753 to 2011. *Geoinformatics & Geostatistics: An Overview* **1** (2013).

27 Gent, P.R. et al. The Community Climate System Model Version 4. J. Climate, 24, 4973–4991 (2011).

28 Voldoire, A. et al. The CNRM-CM5.1 global climate model: description and basic evaluation, Clim. Dyn., 40, 2091-2121, DOI:10.1007/s00382-011-1259 (2013).

29 Scinocca, J.F., McFarlane, N.A., Lazare, M., Li, J., and Plummer, D. The CCCma third generation AGCM and its extension into the middle atmosphere. Atmos. Chem. Phys. Discuss. 8(2):7883- 7930 (2008)

30 Hazeleger, W. et al. EC-Earth: A Seamless Earth-System Prediction Approach in Action. Bull. Amer. Meteor. Soc., 91, 1357–1363 (2010)

31 Griffies, S.M. et al. The GFDL CM3 Coupled Climate Model: Characteristics of the Ocean and Sea Ice Simulations. J. Climate, 24, 3520–3544, doi: http://dx.doi.org/10.1175/2011JCLI3964.1 (2011)

32 Dunne, J.P. et al. GFDL's ESM2 Global Coupled Climate–Carbon Earth System Models. Part I: Physical Formulation and Baseline Simulation Characteristics. J. Climate, 25, 6646–6665 (2012)

33 Schmidt, G.A. et al. Present-Day Atmospheric Simulations Using GISS ModelE: Comparison to In Situ, Satellite, and Reanalysis Data. Journal of Climate 19(2):153-192 (2006).

34 Collins, W.J. et al., Development and evaluation of an Earth-System model - HadGEM2, Geoscientific Model Development 4, 1051-1075 (2011).

35 Hardiman, S.C., Butchart, N., Hinton, T.J., Osprey, S.M. and Gray, L. J. The effect of a well resolved stratosphere on surface climate: Differences between CMIP5 simulations with high and low top versions of the Met Office climate model. Journal of Climate. doi: http://dx.doi.org/10.1175/JCLI-D-11-00579.1 (2012)

36 Dufresne, J.-L. et al. Climate change projections using the IPSL-CM5 Earth System Model: from CMIP3 to CMIP5, Climate Dynamics, 40, 9-10, 2123-2165 , doi:10.1007/s00382-012-1636-1 (2013).

37 Watanabe, M. et al. MIROC-ESM 2010: model description and basic results of CMIP5-20c3m experiments Geosci. Model Dev., 4, 845-872 (2011)

38 Watanabe, M. et al. Improved Climate Simulation by MIROC5: Mean States, Variability, and Climate Sensitivity. J. Climate, 23, 6312–6335 (2010)

39 Giorgetta, M.A. et al. "The atmospheric general circulation model ECHAM6 - Model description" Reports to Earth System Science ISSN 1614-1199 (2012)

40 Yukimoto S. et al. The New Meteorological Research Institute Coupled GCM (MRI-CGCM2) Model Climate and Variability. Papers in Meteorology and Geophysics 51(2):47-88, (2001)

41 Bentsen, M. et al. The Norwegian Earth System Model, NorESM1-M. Part 1: Description and basic evaluation. Geosci. Model Dev. Discuss., 5, 2843-2931 (2012)

42 Tjiputra, J.F. et al. Evaluation of the carbon cycle components in the Norwegian Earth System Model (NorESM). Geosci. Model Dev. Discuss., 5, 3035-3087 (2012)

43 Xin X., Wu T. and Zhang J. Introductions to the CMIP 5 simulations conducted by the BCC climate system model (in Chinese). Advances in Climate Change Research 4, 41-49 (2012)

44 Volodin, E.M., Dianskii, N.A. and Gusev, A.V. Simulating present-day climate with the INMCM4.0 coupled model of the atmospheric and oceanic general circulations. Izvestiya, Atmospheric and Oceanic Physics, 46, 414-431 (2010)
